# Motorized Two-Wheeled Vehicles Contribute Disproportionately to the Increase in Pandemic-Period Road Traffic Fatalities in New York State

**DOI:** 10.3390/ijerph22121883

**Published:** 2025-12-18

**Authors:** Joyce C. Pressley, Zarah Aziz, Leah Hines, Jancarlos Guzman, Emilia Pawlowski, Michael Bauer

**Affiliations:** 1 Departments of Epidemiology and Health Policy and Management, Columbia University in the City of New York, New York, NY 10032, USA; 2 Department of Epidemiology, Columbia University in the City of New York, New York, NY 10032, USA; zarahfaziz@gmail.com; 3 Bureau of Occupational Health and Injury Prevention, New York State Department of Health, Albany, NY 12237, USA

**Keywords:** motorized micromobility, e-bike, motorcycle, mopeds, mortality, safe system, COVID-19

## Abstract

**Highlights:**

**Public health relevance—How does this work relate to a public health issue?**

- Roadway mortality was declining in New York State before the COVID-19 pandemic.
- Since the pandemic, mortality increased and did not return to pre-pandemic levels as the pandemic receded.

**Public health significance—Why is this work of significance to public health?**

- This work improves our scientific understanding of the factors contributing to the COVID-19 road mortality increase in New York State.
- Access to shared motorized two-wheeled vehicles, such as e-bikes, is proliferating ahead of injury prevention strategies to address the mortality increase.

**Public health implications—What are the key implications or messages for practitioners, policy makers and/or researchers in public health?**

- Improvement of data surveillance systems is needed to better characterize the growth of emerging two-wheeled electric vehicles and vehicle types.
- Identification of motorized two-wheeled vehicles as a significant contributor to the increase in pandemic-era roadway mortality can be used to inform the development of countermeasures related to this transportation mode.

**Abstract:**

**Background:** New York State, like many other states, experienced a significant increase in road traffic deaths during the COVID-19 pandemic that is not fully understood. Our earlier work using the Safe System framework suggests a shift in the distribution of vehicle types that may have contributed to this phenomenon. **Methods:** To further investigate this, variables from the Fatality Analysis Reporting System (FARS) were mapped onto the Safe System framework and used to examine factors associated with motorized two- and three-wheeled vehicle deaths. Two time periods were examined: pre-pandemic (1 April 2017–31 December 2019, *n* = 428) and the COVID-19 pandemic era (1 April 2020–31 December 2022, *n* = 600). A buffer pandemic transition period (1 January 2020–31 March 2020) was excluded. Percent difference, chi-square tests, and multivariable logistic regression (OR, 95% CI) were used. **Results:** Compared to pre-COVID-19, pandemic-period motorized two-wheeled deaths were 40.2% higher, helmet wearing lower (80.2% vs. 90.6%, *p* < 0.0001), urban roadway deaths higher (76.7% vs. 64.0%, *p* < 0.0001), and fully licensed drivers lower (78.4% vs. 89.9%, *p* < 0.0001), with unlicensed drivers doubling between the two periods (8.7% to 17.6%, *p* < 0.0001). Deaths associated with mopeds/motor scooters/minibikes increased 361.5% between study periods, from 3% to 10% of motorized two-wheeled deaths. Adjusted multivariable risk factors for pandemic-period death were age 30–39 years (1.601, 1.155–2.311), being unhelmeted (3.191, 2.109–4.968), being in an urban area (1.898, 1.425–2.533), being unlicensed (1.968, 1.228–33.216) and riding an off-road motorcycle (3.753. 1.391–13.063), moped or motor scooter/minibike (3.540, 1.971–6.842). **Conclusions:** Total mortality was higher in the COVID-19–era timeframe, with the increase differing significantly by vehicle type, helmet use, licensure status, and urbanization. Due to the increase in motorized two-wheeled vehicles, they should be incorporated into surveillance systems and injury prevention strategies.

## 1. Introduction

Since the widespread increase in road traffic deaths associated with the COVID-19 pandemic, many states, including New York State (NYS), continue to seek a better understanding of the root causes to support effective countermeasures [1,2,3,4,5,6,7]. While using the five pillars of the Safe System approach to investigate factors associated with this increase, we observed a significant shift in the distribution of road users dying on NYS roadways [8]. This shift included a disproportionate mortality increase among riders of motorized two- and three-wheeled vehicles [8].

The vehicle types in the motorized two-wheeled category are continuing to expand in the United States (U.S.) and globally. In the U.S., this is fueled in part by a desire to commute using more climate-friendly transportation modes [9,10,11,12,13,14]. Although motorized vehicle ownership in the U.S. continues to be primarily four-wheeled vehicles, two-wheeled vehicles are the predominant household vehicle owned in several Asian, African and South American emerging economies primarily due to their having lower purchase, operating and maintenance costs. Two- and three-wheeled vehicles contribute to 30% of global road traffic deaths, ranging as high as 46% of traffic mortality in Southeast Asia [14]. Their widespread use in these countries provides insight into factors impacting ridership, crashes, injury and mortality.

Before the COVID-19 era, e-scooter use was on the rise in the U.S., with shared e-scooter trips reportedly increasing by more than 100% between 2018 and 2019, with a staggering 86 million trips reported [10]. The arrival of COVID-19 and a desire for a socially distanced commute fueled growth in this transportation mode. Federal funding expanded the availability of shared vehicles in this category [15,16,17]. Legislation in many areas is moving these vehicles off sidewalks and onto streets where they share the roadway with larger, heavier vehicles [18]. Given the growth in this transportation mechanism, this study sought to expand our knowledge and understanding of the factors associated with rider injury and mortality.

This study investigates the factors hypothesized to be associated with the increase in pandemic-era mortality in riders of two- and three-wheeled vehicles in NYS. Pre-COVID-19 and COVID-19–era fatal crashes are investigated across the five pillars of the Safe System: (1) road users, (2) vehicles, (3) roadways, (4) speed and (5) post-crash care [19,20,21]. Additional scientific inquiry into factors associated with increased COVID-19–era mortality could yield important scientific insights to inform future prevention efforts. In particular, we aim to characterize behavioral, vehicle, roadway and post-crash care factors associated with the change in mortality between the two study periods.

## 2. Materials and Methods

The Fatality Analysis Reporting System (FARS) [22,23] is used to investigate variables hypothesized to be associated with the reported increase in motorized two- and three-wheeled vehicle mortality in NYS (*n* = 1028) pre-pandemic (1 April 2017 to 31 December 2019, *n* = 428) and in the COVID-19 era (1 April 2020 to 31 December 2022, *n* = 600).

### 2.1. Study Population

The study population included all deaths of drivers and passengers on motorized two- and three-wheeled vehicles traveling on a public roadway in NYS that were captured by FARS [21,22].

The data sources and covariates have been described elsewhere but are repeated here for the convenience of the reader [8]. The study population was categorized into two time periods. The baseline pre-COVID-19 era included fatalities occurring between 1 April 2017 and 31 December 2019 (*n* = 428), while the COVID-19–era included deaths between 1 April 2020 and 31 December 2022 (*n* = 600). A three-month buffer period was used between the pre-COVID era and the COVID era, which excluded roadway deaths from 1 January 2020–31 March 2020.

### 2.2. Data Source(s)

FARS is managed and distributed as a deidentified public-use data set and made available by the National Highway Traffic Safety Administration through internet download [22,23]. The data are a census of all fatal crashes on U.S. roadways and provides information on the person type (driver or passenger), vehicle type and characteristics, roadway features, weather and road conditions, crash characteristics and post-crash emergency transport type and times.

### 2.3. Study Design

The two study periods were designed to capture and compare mortality occurring before and after the emergence of COVID-19. This cross-sectional study compared the magnitude and characteristics of roadway deaths pre-COVID-19 to the COVID-19 era [18]. The first confirmed case of COVID-19 in NYS occurred on 1 March 2020 [24]. Key variables were selected as indicators of each of the five pillars of the Safe System approach: (1) road users, (2) vehicles, (3) roadways, (4) speed and (5) post-crash care. Seasonality effects were controlled through exact matching of the included months in each study period.

### 2.4. Variable Classifications

*Outcome variable.* Two-wheeled vehicle mortality was compared between the two study periods, pre-COVID (*n* = 428) and the COVID era (*n* = 600).

*Covariates.* Independent variables consisted of several road user, roadway, vehicle, crash, speeding and post-crash care characteristics.

#### 2.4.1. Pillar 1: Road User-Level Characteristics

*Age.* Driver and passenger ages were categorized as ≤19, 20–29, 30–39, 40–64 and 65 years and older. Several age constructs were evaluated for drivers, passengers and all riders, including continuous and 10-year age intervals. A collapsed four-category variable was used in multivariable models due to small cell sizes in some age and vehicle categories.

*Sex*. This variable is recorded in FARS as male, female or unknown.

*Use of protective gear.* The use of a helmet was dichotomous as yes (helmeted) or no (not helmeted).

*Alcohol-involved.* Alcohol was available as a dichotomous variable and was derived from the driver having a positive test or police-reported alcohol involvement [8].

*Driver license.* The license was categorized as full, intermediate GDL/learner’s permit, not licensed or other.

#### 2.4.2. Pillar 2: Vehicle and Vehicle Crash Level Characteristics

*Vehicle type*. Vehicles included two- and three-wheeled motorcycles, off-road motorcycles, mopeds, motor scooters and minibikes. Five deaths were on motorcycles of unspecified type.

*Single or multiple vehicle collisions.* Collision type was categorized as single vehicle, two vehicles or multiple vehicles.

*Collision type.* Collisions used the FARS classifications of not a collision with a motor vehicle in transport, angle, head-on, rear-end, sideswipe or other.

*Vehicle maneuver at time of crash.* Vehicle movement was classified as going straight, negotiating a curve, turning, merging/changing lanes, passing or overtaking and other.

#### 2.4.3. Pillar 3: Roadway Characteristics

Urban/Rural. Urbanization was examined using two methods. FARS data include a dichotomous variable categorized as rural or urban. The second method included analysis of the 2013 classification for Rural Urban Continuum Codes. All NYS counties in the seven Rural Urban Continuum Code categories were collapsed into three categories: metropolitan; non-metropolitan, adjacent to a metropolitan area; non-metropolitan, not adjacent to a metropolitan area [25].

*New York State region.* Three regions of NYS were examined between the two study periods. New York City was comprised of five boroughs (counties): New York, Bronx, Queens, Kings, and Richmond (Staten Island). Long Island was comprised of Suffolk and Nassau counties. Upstate was comprised of the 55 remaining counties not included in New York City or Long Island.

*Traffic way characteristics/lanes.* This variable was categorized as one lane, two or more lanes with one-way traffic, two or more lanes with divided two-way traffic and two or more lanes with two-way traffic not divided.

*Intersection type*. Roadways were characterized as not an intersection; four-way intersection; T or Y intersection configuration; or other.

*Road surface.* Road conditions were categorized as dry; not dry (wet, snow/slush, ice/frost); or other/unreported/unknown.

*Weather*. This variable was categorized as clear conditions; rain; cloudy; or other/unreported/unknown.

*Traffic control devices*. This was categorized as no controls, traffic control signal, stop sign, other signs/signals and unknown/not reported.

*Lighting conditions.* This was categorized as daylight; dark, lighted; dark, not lighted; dawn; or dusk.

#### 2.4.4. Pillar 4: Speeding

A speed-related crash was dichotomous. Actual miles per hour traveled were not available for analysis.

#### 2.4.5. Pillar 5: Post-Crash Care

*Died at scene.* This variable is categorized by did not die in route or at the scene; died at the crash scene; or died en route to the hospital.

*Transported and mode of transport*. This variable is categorized as not transported for care (DOA); transported by ground ambulance/fire/police; or by air ambulance.

### 2.5. Statistical Analysis

Variables representing each of the five pillars of the Safe System approach were obtained from FARS 2017–2022. Seasonality was controlled by exact matching on the month of the fatal crash. A transition period between the pre-COVID-19 and COVID-19 era excluded 3 months (1 January 2020 to 31 March 2020). 1 April 2020 comprised the beginning of Time Period 2, the pandemic era. Data representing each of the five pillars of the Safe System were examined for numerical characteristics using bivariable analyses before being used in multivariable models. Chi-square tests and unadjusted and adjusted multivariable logistic regression were used to analyze data. Our objective was to investigate characteristics of fatalities using the Safe System before COVID-19 compared to the COVID-19 era. Binominal logistic regression models were constructed to examine differences in mortality risk factors between the pre-COVID-19 and COVID-19 eras using the variables mapped across the Safe System framework as the independent variables [26]. Unadjusted models were examined by sequentially placing a single variable of interest into the logistic model and recording the point estimate and 95% confidence interval for that variable. Subsequent variables were placed into the model one at a time until the unadjusted point estimates had been determined for each variable. Model building proceeded by selecting variables with unadjusted significance equal to lower than 0.20. Several age constructs were evaluated, including continuous, 10-year age intervals and a collapsed version that confirmed that the ages 30–39 years were robust in their increased risk of death. Final adjusted models were sex-adjusted and included variables with a significance of 0.05 or lower. The Hosmer and Lemeshow goodness of fit test was used to assess how well the multivariable model fit. This model’s interpretation is that larger *p* values indicate that the model fits well, with small *p*-values of less than 0.05 indicating that it is a poor fit. All bivariable statistical analyses were two-sided, and a *p*-value ≤ 0.05 was considered statistically significant. All analyses were performed in R [27,28].

## 3. Results

**Figure 1:**
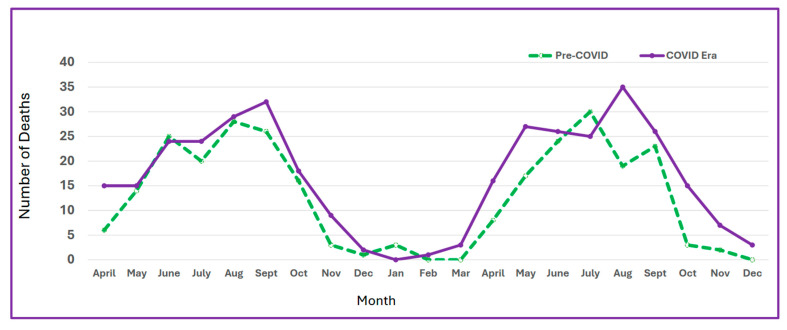
Percent mortality by month of motorized two- and three-wheeled vehicle riders in the pre-COVID era (green dotted line) and in the COVID era (purple line), FARS data, 2018–2021.

Total mortality on NYS roadways was higher during the COVID-19 era compared to the pre-COVID-19 time period, with 40.2% of the total mortality increase occurring in riders of motorized two- and three-wheeled vehicles (Table 1). The increase differed significantly across pillars of the Safe System and across seasons (Figure 1).

### 3.1. Pillar 1: Road User Characteristics

*Age/Sex.* Mortality is shown by age group and by sex in Table 1. The age group experiencing the largest percentage increase in mortality was adults aged 30–39 years, where mortality nearly doubled in the COVID-19 era compared to pre-COVID-19. Of note, riders aged 65 and over comprised only 6.2% of all pre-COVID-19–era riders but had an 80% increase in ridership deaths during the COVID-19 era (Table 1). Total mortality by time period is shown for males and females in Table 1. Both males and females experienced higher mortality during the COVID-19 era, but differences were not statistically significant (Table 1).

*Road user type*. Drivers experienced a 41.8% higher mortality, with passengers increasing by a smaller 12.5% (see Supplementary Table). Male drivers comprised approximately 92% of mortality in both study periods. Males rarely died as passengers compared to females (0.6% vs. 4.4%). Females tended to die as passengers more frequently than as drivers (2.9% vs. 4.4%). Sex differences were not significantly different between the two study periods (Table 1).

*Helmet use.* Compared to the pre-pandemic era, helmet wearing in all riders was lower during the pandemic (90.6% vs. 80.2%, *p* < 0.0001). Mortality increased in both helmeted and helmetless riders during the pandemic. However, the percentage increase for helmeted riders during the pandemic was higher than the pre-pandemic era by 25.3% compared to 270.4% for riders who were helmetless (*p* < 0.0001) (Table 1).

*Licensure.* Pre-COVID-19, 89.9% of motorized two and three-wheeled driver deaths occurred in fully licensed drivers, but this was lower (78.4%) during the pandemic (*p* < 0.0001). As a proportion of all motorized two- and three-wheeled driver deaths, unlicensed driver deaths doubled from 8.7% to 17.6%. The intermediate or learner’s permit licensure category was a small number of deaths but quadrupled during the pandemic to a total of 17, with this category rising from 1.0% to 3.0% of all driver deaths.

*Impairment.* No significant difference was recorded in alcohol-related impairment between the pre-pandemic and pandemic eras (Table 1).

### 3.2. Pillar 2: Vehicle Characteristics

Several vehicle and crash features were compared between the two study periods (Table 1). More than one-third of crashes pre-pandemic were single-vehicle collisions. The largest change between the two study periods occurred in multiple (2+) vehicle crashes, which more than doubled during the pandemic era.

*Vehicle type*. Crashes involving mopeds, scooters, minibikes, and off-road motorcycles increased by more than 300% between the two study periods (*p* < 0.0001) (Table 1). Absolute fatalities of riders of these vehicles increased (*n* = 17 to 79, *p* < 0.0001).

*Vehicle crash characteristics*. Although overall collision type did not differ significantly between the two study eras, it is notable that approximately three-quarters of all fatalities were accounted for by two collision categories (Table 1). Rear-end collisions comprised one-third of all fatalities, and not a collision with a motor vehicle in transport accounted for approximately 40%. Sideswipes comprised only 3.3% of all crashes (14 deaths) pre-pandemic but approximately doubled during the pandemic (29 deaths) (Table 1).

Vehicle maneuver at time of crash did not differ between the two study periods with two categories of vehicle maneuver at time of crash (going straight and negotiating a curve) accounting for approximately 85% of all deaths in both study periods. Vehicle passing or overtaking accounted for approximately 6–7% of vehicle crashes in both study periods (Table 1).

### 3.3. Pillar 3: Roadway Characteristics

Roadway characteristics are shown in Table 2. Several characteristics were associated with increased mortality during the COVID-19 era compared to pre-COVID.

*Urbanization.* Using the urban–rural designation in FARS, urban roadways accounted for nearly two-thirds of motorized two- and three-wheeler deaths pre-COVID-19, but this increased to more than three-fourths of deaths in the COVID-19 era (*p* < 0.0001). When using the RUCC categorization, deaths in metropolitan areas increased nearly 50% during the COVID-19 era, accounting for 90% of all fatalities in the pandemic era (*p* = 0.012). Deaths in both non-metropolitan area categories decreased, with the largest decrease occurring in metropolitan areas not adjacent to a metropolitan area (Table 2).

*New York State Region.* Death counts on motorized two-wheelers increased in all three regions of NYS during the COVID-19 era, but differences between the three regions were not statistically different (*p* = 0.325). Approximately 60% of motorized two- and three-wheeler deaths during both study periods occurred in the 55-county Upstate region. The percentage increase in mortality tended to be highest in New York City, with Long Island experiencing the smallest increase during the COVID-19 era (Table 2). During the pre-COVID-19 era, New York City accounted for 22.5% of statewide motorized two- and three-wheeler deaths, but this increased by more than 60% during the COVID-19 era to 26.1% of all deaths (Table 2).

Approximately 68% of deaths occurred on two-lane roads with non-divided two-way traffic. This road characteristic did not differ between the two study periods (Table 2). Approximately 95% of crashes in the total study population occurred on dry roads, and 79% occurred during clear weather conditions (*p* = NS) (Table 2). Other roadway characteristics with non-significant differences between the two study eras included lighting conditions and traffic control devices (Table 2).

Approximately three-quarters of all deaths occurred in areas without any traffic control devices (Table 2). More than 60% of crashes occurred on a road segment categorized as not an intersection, with non-intersection crashes tending to be higher during the COVID-19 era (65.3% vs. 60.3%, *p* = 0.074). Deaths occurring on non-intersection road segments tended to increase more during the pandemic than deaths at four-way, T or Y intersections (Table 2).

### 3.4. Pillar 4: Speeding

Motorized two-wheeler driver deaths characterized as speed-related did not differ between the two study periods (*p* = 0.901) (Table 1).

### 3.5. Pillar 5: Post Crash Care

*Mode of transport.* The number of crash victims who were not transported to a hospital increased by 62.2% during the COVID-19 era (*p* = 0.088). The proportion of all crash victims not transported increased from 24.0% to 36.5% during the pandemic. Transport by air ambulance was rare pre-COVID-19 (*n* = 6) but more than doubled during the pandemic (*n* = 15) (Table 2).

### 3.6. Independent Risk Factors for Mortality for Persons on Motorized Two- and Three-Wheeler Vehicles

**Figure 2:**
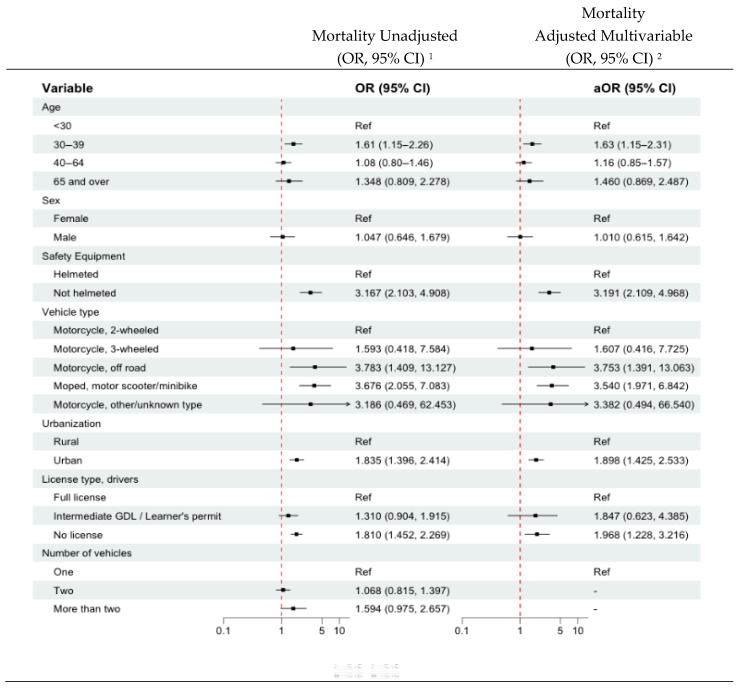
Unadjusted and adjusted multivariable risk factors for mortality on motorized two- and three-wheeled vehicles in the COVID-19 era compared to pre-COVID-19, FARS 2017–2022. ^1^ Unadjusted consisted of each variable being entered into the model one at a time. ^2^ Model building strategy included adding in all unadjusted variables significant at the 0.20 level and retention of those significant at the 0.05 level. Table S1 is included in the Supplementary File section and includes the variable name, point estimate, 95% CI and the *p* value for significance.

Risk factors for mortality during the COVID-19 era are shown in Figure 2 for unadjusted and adjusted independent variables. Additional information on the multivariable model variables is shown in the Supplemental File. Variables across all five pillars of the Safe System framework were modeled using logistic regression and are shown with 95% CI in Figure 2. Riders aged 30–39 years had 63% higher odds of mortality than younger riders aged less than 30 years (OR 1.630, 95% CI 1.155, 2.311). When examining the urbanicity of roadways, urban crash mortality was 90% higher (OR 1.898, 95% CI 1.425, 2.533). In the COVID-19 era, the odds of mortality were significantly higher in riders who were not wearing a helmet (OR 3.191, 95% CI 2.109, 4.968). Within the motorized two and three wheelers, vehicle type was a risk factor for mortality. Two vehicle categories were associated with higher mortality. Use of the moped/motor scooter/minibike was higher during the pandemic era compared to pre-pandemic (OR 3.540, 95% CI 1.971, 6.842) as was use of off-road motorcycles (OR 3.753 95% CI 1.391, 13.063). Failure to be licensed to drive was a risk factor associated with higher mortality in adjusted models in the COVID-19 era. Deaths were 97% higher in drivers with no license in the COVID-19 era (OR 1.968, 95% CI 1.228, 3.216) (Figure 2). Interaction terms between vehicle types (many of which had small cell sizes) and other covariates were not significant. The Hosmer and Lemeshow goodness of fit test showed X2-4.4539, df = 8 with a *p*-value of 0.814, which indicates a reasonably good model fit.

## 4. Discussion

The pandemic was accompanied by a significant increase in road traffic mortality in NYS [8,29]. As the pandemic receded, the increase in NYS roadway mortality remained elevated and did not return to its pre-COVID-19 declining mortality trajectory. In an earlier study that included all road users in NYS, we found that approximately 40% of the COVID-19–era excess roadway mortality occurred in more vulnerable road user types—motorized two- and three-wheeled vehicles [8]. Examination of this shift in mortality identified significant factors associated with the increase in three of the Safe System’s five pillars (road users, vehicles and roadways) with two other pillars (speed and post-crash care) showing non-significant changes. Compared to the pre-COVID-19 era, pandemic era fatalities were significantly less likely to have been wearing protective headgear, less likely to be fully licensed drivers and more likely to die on an urban roadway. In the pre-COVID-19 era, motor vehicle licensure and registration offices were open for business. During the pandemic era, the offices responsible for licensure and vehicle registration were closed. In addition, the driver education and training that was required for young drivers during the pre-pandemic era was unavailable during the COVID-19 era.

In a systematic review of helmet law enactment and repeal, helmet wearing increased, and severe head injuries decreased with the enactment of a motorcycle law. This analysis of 60 qualifying U.S. helmet studies showed a 42% reduction in the risk for fatal injuries and a 69% reduction in the risk for head injuries with enactment of the law. However, in geographic areas where helmet laws were repealed, helmet wearing decreased 39 percentage points per registered motorcycle, and total deaths increased by 24% [30].

The findings of this study demonstrate that motorized two- and three-wheeler deaths contribute disproportionately to roadway mortality in NYS, with this increase differing significantly across vehicle types. The largest increase was among the off-road/moped/motor scooter/minibike category. This is consistent with reports in other countries that have a longer history with moped, minibikes and e-scooters on their roadways [15,16,17,18,19].

The U.S. government funded cities and urban areas to increase the availability of shared electric scooters and motorized two-wheeled bicycles, whose riders may not be subject to the same laws that apply to larger, more powerful motorcycles [31]. Having less stringent safety equipment laws for less powerful motorized two-wheeled vehicles has been shown to be associated with higher mortality and more severe head injuries compared to more regulated motorcycles [32,33]. This finding was in an environment where the safety equipment laws did not apply to smaller, less powerful motorized two-wheeled vehicles [32,33]. Other countries that have decades more experience than the U.S. with motorized two-wheeled vehicle types have shown that traumatic brain injury and other severe injuries differ across various safety regulations [34]. In a large study of 33,400 motorcycle, moped and light moped crashes, including 19,700 moped crashes and more than 3000 light moped users, light moped users in jurisdictions without mandatory helmet laws were more likely to sustain severe head injuries and to die [34]. Our study also found that a lower proportion of moped/scooter drivers were licensed compared to motorcycle drivers.

Roadway characteristics differed significantly between the two study periods, with larger mortality increases observed on urban than on rural roads. Enforcement of traffic laws was lower in incorporated areas than in more rural areas [35]. Further study is needed to assess countermeasures to accompany the exponential growth in motorized two-wheeled transportation alternatives, particularly in urban areas where the growth is strongest.

While speeding was not recorded as significantly different between the two study eras, enforcement in NYS was lower during the pandemic era compared to the pre-COVID-19 era. In that context, there was higher mortality at non-intersections compared to intersections, consistent with higher speeds on non-intersection segments of the roadway. Across the two study periods, the majority of deaths occurred in males. Increases tended to be higher in those aged 30–39 years and in those over age 65 years, with no sex differences noted between the two study periods. This is consistent with studies of motorized two-wheeled vehicles in other countries that have observed higher injury and mortality in young males [34,36,37].

Post-crash care characteristics tended to differ between the two study periods, with more deaths occurring at the roadside and fewer riders transported for post-crash care. This is consistent with our findings of an increase in more road users riding helmetless and experiencing more severe injuries, as indicated by more deaths at the scene of the crash. Among those who were transported for emergency department/hospital care, response and travel times tended to be shorter than pre-pandemic times, possibly due to lower traffic volumes during the pandemic [8].

NYS has worked simultaneously to increase the accessibility of shared electric mobility while recognizing and addressing the fact that the technology has outpaced our legislation and provision of countermeasures [38,39]. The promotion of thoughtful e-scooter legislation and infrastructure changes has been reported to help promote safer travel [40].

Since this study data were collected, NYS has passed two new policy countermeasures, with numerous others introduced. Currently, a new law requires registration of these vehicles at the point of sale. Additionally, a new regulation requires improved surveillance related to the type and use of the vehicles [41]. Additional workgroups are currently addressing improvements needed to classify these and other evolving unusual vehicle types. New York City has passed a 15-mile-per-hour speed limit for e-bike vehicles.

Several countermeasures are associated with decreased injury in this population. Stronger helmet laws are reported to be associated with higher incidence of helmet wearing at the time of crash and with lower costs for post-crash care for those who survive to hospital admission [42,43]. In states with universal helmet laws, on average, 12% of fatally injured motorcyclists were not wearing helmets compared to 79% in states without a helmet law. States with partial helmet law coverage, such as laws that only required specific groups, such as young riders, to wear helmets, were intermediate, with 64% riding without a helmet at the time of the fatal crash. Societal cost savings in states with a universal helmet law were nearly four times greater than in states without such a law [42]. Dua et al. conducted a cost analysis of helmetless motorcycle riders and concluded that per capita inpatient and indirect costs were more than $800,000, culminating in nearly $2.2 billion in societal losses per year [43].

Other countermeasures that have been shown to be effective for both those using motorized two- and three-wheeled vehicles and those sharing the road with them include regulatory approaches for vehicle inspections, licensure of drivers, and enforcement of speed laws, red-light running, and impaired driving. More recently, mandatory classes on rules of the road, roadway and signage redesign and meaningful penalties, such as vehicle confiscation for violations that endanger others, are still being evaluated.

One limitation of our current study is that it captures injury to drivers and passengers of two- and three-wheeled vehicles but not injuries to other road users associated with the proliferation of these vehicles, such as pedestrians [44]. While existing FARS information is extensive, it is somewhat limited regarding two-wheeled vehicles possibly contributing to misclassification of vehicle types by reporting officers. Surveillance improvements are needed to standardize two-wheeled vehicle categorizations in FARS. A pictorial card of two-wheeled types for use by officers would allow more consistent categorization of vehicles. Several vehicle types have become more prevalent on the roadway since the last update of FARS. Many jurisdictions have adopted such pictorial categorizations, but these do not yet appear in FARS. Future research is needed to investigate ways to increase access to helmets for riders of shared vehicles.

Law enforcement efforts, as measured by citation rates during the COVID-19 pandemic, were lower than pre-pandemic, which may have impeded our ability to detect the influence of potentially contributing behaviors, such as speeding and alcohol, in the pandemic era. When captured, speed was a dichotomous variable and did not indicate miles per hour. Detection of drug impairment has been noted to be a limitation of FARS in general [45], which would have had an impact on both study periods. Mortality that occurred after 30 days is not captured in FARS. It is possible that FARS undercounts deaths related to motorized two-wheeled vehicles given that FARS collects data on deaths on U.S. roadways within 30 days of the crash and does not report deaths that occur on these vehicles in other locations, such as on private property or in parks. We did not have data on possible road infrastructure changes that occurred during the study. COVID-19 travel patterns and travel volume varied across the state and across time periods. We were unable to assess exposure related to traffic volume, vehicle miles traveled or the influence of temperature fluctuations that have been demonstrated to influence mortality in other settings [46]. In NYS, pandemic lockdowns began in March of 2020, with New York City public schools shutting down on 16 March. The lockdowns occurred at various times across the state, all beginning in March 2020. The buffer period excludes eight deaths that occurred between January and March of 2020 [46,47].

A strength of the study is that it addressed the significant seasonal variation using a study design that exactly matched months and the length of pre-COVID-19 and COVID-19 eras.

## 5. Conclusions

In conclusion, NYS, like many other states, experienced a significant increase in road traffic deaths during the COVID-19 pandemic [8,32,33,34,35,36]. Our finding of a disproportionate increase in deaths associated with more vulnerable two-wheeled vehicles suggests that the growth and popularity of two-wheeled vehicles is a contributor to the reversal of the downward trends in roadway mortality in NYS. Compared to the pre-COVID-19 era, pandemic era fatalities were significantly less likely to have been wearing protective headgear, less likely to be fully licensed drivers and more likely to die on an urban compared to a rural roadway. The exponential growth of this mode of transportation in urban areas suggests the need for innovative countermeasures to address the increased proliferation of these vehicles without accompanying safety measure provisions.

Further study is needed to address lapses in surveillance, issues in data quality with regard to categorization of vehicle types and gaps in safety regulations, driver training, licensure, enforcement, public education and on-site availability of helmets for shared rental vehicles. Motorized two-wheeled rider crash death is an emerging and growing issue that should be incorporated into our guidance documents and surveillance systems [47].

## Figures and Tables

**Table 1:** Road user, speed and vehicle characteristics for fatal crashes in motorized two- and three-wheeled vehicles pre-COVID and in the COVID era in New York State, FARS 2017–2022 ^1^.

		Mortality Pre-COVID	Mortality COVID Era	Total Mortality		
	% Change	1 April 2017–31 December 2019	1 April 2020–31 December 2022	1 April 2017–31 December 2022	Chi-square	
		n (%)			X^2^ (*p*-value)	
*n* (%)	40.2	*n* = 428	*n* = 600	*n* = 1028 ^1^		
**Pillar I: Rider characteristics**						
Type		428	600	1028	0.436 (0.509)	
Drivers	41.8	404 (94.4)	573 (95.5)	977 (95.0)		
Passengers	12.5	24 (5.6)	27 (4.5)	51 (5.0)		
Age, Drivers (years)		404	571	975	13.442 (0.062)	
≤19	21.4	14 (3.5)	17 (3.0)	31 (3.2)		
20–29	14.3	140 (34.7)	160 (28.0)	300 (30.8)		
30–39	92.7	82 (20.3)	158 (27.7)	240 (24.6)		
40–64	33.6	143 (35.4)	191 (33.5)	334 (34.3)		
65 and over	80.0	25 (6.2)	45 (7.9)	70 (7.2)		
Sex		428	600	1028	0.004 (0.947)	
Male	40.7	396 (92.5)	557 (92.8)	953 (92.7)		
Drivers	41.0	393 (91.8)	554 (92.3)	947 (92.1)		
Passengers	0.0	3 (0.7)	3 (0.5)	6 (0.6)		
Female	34.4	32 (7.5)	43 (7.2)	75 (7.3)		
Drivers	72.7	11 (2.6)	19 (3.2)	30 (2.9)		
Passengers	14.3	21 (4.9)	24 (4.0)	45 (4.4)		
Helmet, wearing						
Total study population (n)		424	600	1024	23.478 (<0.0001)	
No	270.4	27 (6.4)	100 (16.7)	127 (12.4)		
Yes	25.3	384 (90.6)	481 (80.2)	865 (84.5)		
Unknown	46.2	13 (3.1)	19 (3.2)	32 (3.1)		
Drivers (n)		400	573	973	20.744 (<0.0001)	
No	268.0	25 (6.3)	92 (16.1)	117 (12.0)		
Yes	27.3	363 (90.8)	462 (80.6)	825 (84.8)		
Unknown	58.3	12 (3.0)	19 (3.3)	31 (3.2)		
Alcohol-involved, Drivers		404	573	977	1.845 (0.174)	
No	49.8	291 (72.0)	436 (76.1)	727 (74.4)		
Yes	21.2	113 (28.0)	137 (23.9)	250 (25.6)		
License Type, Drivers		404	573	977	21.719 (<0.0001)	
Full license	23.7	363 (89.9)	449 (78.4)	812 (83.1)		
Intermediate GDL/Learner’s Permit ^2^	325.0	4 (1.0)	17 (3.0)	21 (2.1)		
No License	188.6	35 (8.7)	101 (17.6)	136 (13.9)		
Other	200.0	2 (0.5)	6 (1.0)	8 (0.8)		
**Pillar II: Vehicle Characteristics**						
Vehicle type		428	600	1028	25.605 (<0.0001)	
Motorcycle, 2-wheeler	25.6	407 (95.1)	511 (85.2)	918 (89.3)		
Motorcycle, 3-wheeler	100.0	3 (0.7)	6 (1.0)	9 (0.9)		
Motorcycle, off-road	375.0	4 (0.9)	19 (3.2)	23 (2.2)		
Moped, motor scooter/minibikes	361.5	13 (3.0)	60 (10.0)	73 (7.1)		
Motorcycle, unknown type	300.0	1 (0.2)	4 (0.7)	5 (0.5)		
Number of Vehicles		428	600	1028	3.411 (0.182)	
Single	29.93	147 (34.3)	191 (31.8)	338 (32.9)		
Two	38.74	253 (59.1)	351 (58.5)	604 (58.8)		
Multiple (2+)	107.14	28 (6.5)	58 (9.7)	86 (8.4)		
Collision type		428	598	1026	1.947 (0.856)	
Not a collision with MV in transport	38.51	174 (40.7)	241 (40.3)	415 (40.4)		
Angle	25.58	43 (10.0)	54 (9.0)	97 (9.5)		
Head-on	38.00	50 (11.7)	69 (11.5)	119 (11.6)		
Rear-end	38.62	145 (33.9)	201 (33.6)	346 (33.7)		
Sideswipe	107.14	14 (3.3)	29 (4.8)	43 (4.2)		
Other	100.00	2 (0.5)	4 (0.7)	6 (0.6)		
Vehicle maneuver at crash		428	600	1028	7.286 (0.506)	
Going straight	44.8	248 (57.9)	359 (59.8)	607 (59.0)		
Negotiating a curve	24.0	125 (29.2)	155 (25.8)	280 (27.2)		
Turning/changing/merging lanes	33.3	21 (4.9)	28 (4.7)	45 (4.4)		
Passing or overtaking	53.9	26 (6.1)	40 (6.7)	66 (6.4)		
Other	125.0	8 (1.9)	18 (3.0)	26 (2.5)		
**Pillar IV: Speed**						
Speed-related crash		428	600	1028	0.016 (0.901)	
No	41.7	235 (54.9)	333 (55.5)	568 (55.3)		
Yes	38.3	193 (45.1)	267 (44.5)	460 (44.7)		

^1^ Total column numbers may not sum to the full population due to missing data. ^2^ GDL Graduated driver license.

**Table 2:** Mortality in motorized two- and three-wheeler fatal crashes pre-COVID and in the COVID-19 era in New York State by roadway, vehicle, crash and post-crash characteristics, FARS 2017–2022 ^1^.

		Mortality Pre-COVID	Mortality COVID Era	Total Mortality ^1^		
	% Change	1 April 2017–31 December 2019	1 April 2020–31 December 2022	1 April 2017–31 December 2022	Chi-square	
		n (%)			X^2^ (*p*-value)	
**Pillar III: Roadway Characteristics**						
Urbanization					18.502 (<0.0001)	
Rural	−8.50	153 (35.7)	140 (23.3)	293 (28.5)		
Urban	67.88	274 (64.0)	460 (76.7)	734 (71.4)		
RUCC rankings					8.878 (0.012)	
Metropolitan	49.17	362 (84.6)	540 (90.0)	902 (87.7)		
Non-metropolitan, adjacent	−1.72	58 (13.6)	57 (9.5)	115 (11.2)		
Non-metropolitan, non-adjacent	−62.50	8 (1.9)	3 (0.5)	11 (1.1)		
NY State geographical area					2.248 (0.325)	
New York City	63.83	94 (22.5)	154 (26.1)	248 (24.6)		
Long Island	21.13	71 (17.0)	86 (14.6)	157 (15.6)		
Upstate	38.89	252 (60.4)	350 (59.3)	602 (59.8)		
Number of lanes					1.528 (0.676)	
One	86.67	15 (3.5)	28 (4.8)	43 (4.3)		
Two or more, one-way traffic	0.00	12 (2.8)	12 (2.1)	24 (2.4)		
Two or more, two-way traffic, divided	38.10	105 (24.8)	145 (24.8)	250 (24.8)		
Two or more, two-way traffic, not divided	37.46	291 (68.8)	400 (68.4)	691 (68.6)		
Intersection type					6.926 (0.074)	
Not an intersection	51.94	258 (60.3)	392 (65.3)	650 (63.2)		
Four-way intersection	35.56	90 (21.0)	122 (20.3)	212 (20.6)		
T and Y intersections	3.75	80 (18.7)	83 (13.8)	163 (15.9)		
Road surface					5.589 (0.133)	
Non-trafficway area/driveway access	--	0 (0.0)	1 (0.2)	1 (0.1)		
Dry	42.22	405 (94.6)	576 (96.0)	981 (95.4)		
Wet, water	−28.57	21 (4.9)	15 (2.5)	36 (3.5)		
Other/not reported/unknown	250.00	2 (0.5)	8 (1.3)	10 (1.0)		
Weather					5.492 (0.139)	
Clear conditions	46.36	330 (77.1)	483 (80.5)	813 (79.1)		
Rain	−40.00	10 (2.3)	6 (1.0)	16 (1.6)		
Cloudy	27.38	84 (19.6)	107 (17.8)	191 (18.6)		
Fog/smog/smoke/crosswinds, other	−66.67	4 (0.1)	4 (0.07)	8 (0.07)		
Traffic control devices					7.708 (0.103)	
No controls	31.45	337 (78.7)	443 (73.8)	780 (75.9)		
Traffic control signal	59.38	64 (15.0)	102 (17.0)	166 (16.1)		
Stop sign	−50.00	12 (2.8)	6 (1.0)	18 (1.8)		
Yield sign	--	0 (0.0)	1 (0.2)	1 (0.1)		
Other signs/signals	--	0 (0.0)	2 (0.3)	2 (0.2)		
Unknown/not reported	206.67	15 (3.5)	46 (7.7)	61 (5.9)		
Lighting conditions					3.067 (0.216)	
Daylight	43.21	243 (56.8)	348 (58.0)	591 (57.5)		
Dark, not lighted	26.00	50 (11.7)	63 (10.5)	113 (11.0)		
Dark, lighted	52.94	102 (23.8)	156 (26.0)	258 (25.1)		
Dawn	25.00	4 (0.9)	5 (0.8)	9 (0.9)		
Dusk	−14.29	28 (6.5)	24 (4.0)	52 (5.1)		
Unknown/not reported	300.00	1 (0.2)	4 (0.7)	5 (0.5)		
**Pillar V: Post Crash Care**						
DOA ^2^				1028	1.733 (0.188)	
Not dead at scene or en route	31.6	291 (68.0)	383 (63.8)	674 (65.6)		
Yes, DOA	58.4	137 (32.0)	217 (36.2)	354 (34.4)		
Dead at scene	60.4	134 (97.8)	215 (99.1)	349 (98.6)		
Dead en route	−33.3	3 (2.2)	2 (0.9)	5 (1.4)		
Mode of transport					4.863 (0.088)	
Not transported	62.22	135 (24.0)	219 (36.5)	354 (30.4)		
Ambulance, ground	26.83	287 (51.0)	364 (60.7)	651 (56.0)		
Ambulance, air	150.00	6 (1.1)	15 (2.5)	21 (1.8)		

^1^ Numbers may not equal column totals due to missing data. ^2^ DOA, Dead on arrival.

## Data Availability

Data are available on the Fatality Analysis Reporting System (FARS) website, https://www.nhtsa.gov/research-data/fatality-analysis-reporting-system-fars (accessed on 20 January 2025), distributed for free by the NHTSA.

## Supplementary Materials

The following supporting information can be downloaded at: https://www.mdpi.com/article/10.3390/ijerph22121883/s1, Table S1: Unadjusted and adjusted multivariable risk factors for mortality on motorized two- and three-wheeled vehicles during the COVID-19 era compared to pre-COVID-19, FARS 2017-2022.

## Abbreviations

The following abbreviations are used in this manuscript:

NYS	New York State
FARS	Fatality Analysis Reporting System
U.S.	United States
GDL	Graduated driver license
