# Supplementary material for: Motorized Two-Wheeled Vehicles Contribute Disproportionately to the Increase in Pandemic-Period Road Traffic Fatalities in New York State
Source: Int J Environ Res Public Health. 2025 Dec 18;22(12):1883. doi: 10.3390/ijerph22121883 (PMC12732951; doi:10.3390/ijerph22121883)
Supplement: Supplementary file 1 [file ijerph-22-01883-s001.zip › ijerph-3974971-supplementary.pdf]

Table S1. Unadjusted and adjusted multivariable risk factors for mortality on motorized two- and three-wheeled vehicles in the COVID-19 era compared to pre-COVID-19, FARS 2017-2022

	Mortality Unadjusted (OR, 95% CI)[1]	Mortality Adjusted Multivariable (OR, 95% CI)[2]
**Pillar I**		
Age (Years)		
<30	Ref	Ref
30-39	1.607 (1.150, 2.256)	1.630 (1.155, 2.311)
40-64	1.082 (0.804, 1.456)	1.156 (0.851, 1.571)
65 and over	1.348 (0.809, 2.278)	1.460 (0.869, 2.487)
Sex		
Female	Ref	Ref
Male	1.047 (0.646, 1.679)	1.010 (0.615, 1.642)
Safety Equipment		
Helmeted	Ref	Ref
Not helmeted	3.167 (2.103, 4.908)	3.191 (2.109, 4.968)
**Pillar II**		
Vehicle type		
Motorcycle, 2-wheeled	Ref	Ref
Motorcycle, 3-wheeled	1.593 (0.418, 7.584)	1.607 (0.416, 7.725)
Motorcycle, off road	3.783 (1.409, 13.127)	3.753 (1.391, 13.063)
Moped, motor scooter/minibike	3.676 (2.055, 7.083)	3.540 (1.971, 6.842)
Motorcycle, other/unknown type	3.186 (0.469, 62.453)	3.382 (0.494, 66.540)
**Pillar III**		
Urbanization		
Rural	Ref	Ref
Urban	1.835 (1.396, 2.414)	1.898 (1.425, 2.533)
License Type, Drivers		
Full license	Ref	Ref
Intermediate GDL / Learner's permit	1.310 (0.904, 1.915)	1.847 (0.623, 4.385)
No license	1.810 (1.452, 2.269)	1.968 (1.228, 3.216)
Number of vehicles		
One	Ref	-
Two	1.068 (0.815, 1.397)	-
More than two	1.594 (0.975, 2.657)	-
Number of lanes		
One	Ref	-
Two or more, one way traffic	0.536 (0.191, 1.480)	-
Two or more, two way traffic, divided	0.740 (0.368, 1.436)	-
Two or more, two way traffic, not divided	0.736 (0.377, 1.384)	-
Intersection type		
Not an intersection	Ref	-

Four-way intersection	0.892 (0.652, 1.224)	-
T and Y intersections	0.683 (0.483, 0.965)	-
Other	N/A	-
Weather		
Clear conditions	Ref	-
Rain	0.410 (0.138, 1.115)	-
Cloudy	0.870 (0.634, 1.198)	-
Sleet/Hail, Snow, Freezing Rain/Drizzle	N/A	-
Fog/Smog/Smoke, Crosswinds, Other	0.228 (0.011, 1.787)	-
Unknown/not reported	2.050 (0.261, 41.535)	-
Traffic control devices		
No controls	Ref	-
Traffic control signal	1.212 (0.862, 1.715)	-
Stop sign	0.380 (0.131, 0.990)	-
Yield sign	N/A	-
Railway crossing	N/A	-
Other signs/signals	N/A	-
Unknown/not reported	2.333 (1.311, 4.386)	-
Lighting conditions		
Daylight	Ref	-
Dark, not lighted	0.880 (0.587, 1.324)	-
Dark, lighted	1.068 (0.793, 1.442)	-
Dawn	0.873 (0.229, 3.557)	-
Dusk	0.599 (0.336, 1.057)	-
Unknown/not reported	2.793 (0.410, 54.819)	-
**Pillar V**		
DOA		
Not dead at scene or en route	Ref	-
Yes, DOA	1.203 (0.926, 1.567)	-
Mode of transport		
Not transported	Ref	-
Ambulance, ground	0.782 (0.599, 1.018)	-
Ambulance, air	1.541 (0.610, 4.409)	-
Fire/police	N/A	-

[1]Unadjusted consisted of each variable being entered into the model one at a time.

[2]Model building strategy included adding in all unadjusted significant variables to test adjusted significance.
